# Clinical Outcomes of Diode Laser Treatment of Ankyloglossia in Children and Young Adults: A Report of Two Cases

**DOI:** 10.7759/cureus.7367

**Published:** 2020-03-22

**Authors:** Pietro Paolo Mezzapesa, Giulia Lepore, Valerio Acella, Nicola De Giglio, Gianfranco Favia

**Affiliations:** 1 Interdisciplinary Department of Medicine, University of Bari Aldo Moro, Bari, ITA; 2 Dentistry, University of Bari Aldo Moro, Bari, ITA

**Keywords:** ankyloglossia, lingual frenotomy, lingual frenectomy, tongue-tie, laser, lingual frenulum

## Abstract

Lingual frenectomy/frenotomy is a relatively safe procedure for removing the lingual frenulum when it is thick, very tight, and/or restricting tongue movements, especially in children. Among all treatment options, diode laser surgery is the most effective. We present two cases wherein diode laser surgery was safe, with a near-total absence of intraoperative bleeding.

## Introduction

Ankyloglossia, also known as tongue-tie, is a relatively rare congenital condition with a prevalence ranging from 4.4% to 4.8% in newborns [1-3]. Although controversy still exists over diagnostic criteria and therapeutic approach, it is generally accepted that ankyloglossia in newborns and children may influence breastfeeding, speech development, dental development, periodontal health, eating, and digestion [4-6]. Several surgical techniques for frenectomy or frenotomy have been reported. The conventional surgical approach is known to be quite invasive and poorly tolerated by patients, unlike laser surgery (diode, potassium titanyl phosphate, neodymium-doped yttrium aluminum garnet [Nd:YAG], erbium:YAG), which represents a real innovation with regard to the absence of intraoperative bleeding, reduction of postoperative edema, unnecessary stitches, and faster mucosal healing [7-9].

We report the two cases: the first case is a child with severe ankyloglossia treated by diode laser frenotomy, highlighting the clinical advantages of this modality. The second case is a young woman with ankyloglossia suffering from dental phobia treated by diode laser frenotomy along with light conscious sedation.

## Case presentation

Case 1

**Figure 1:**
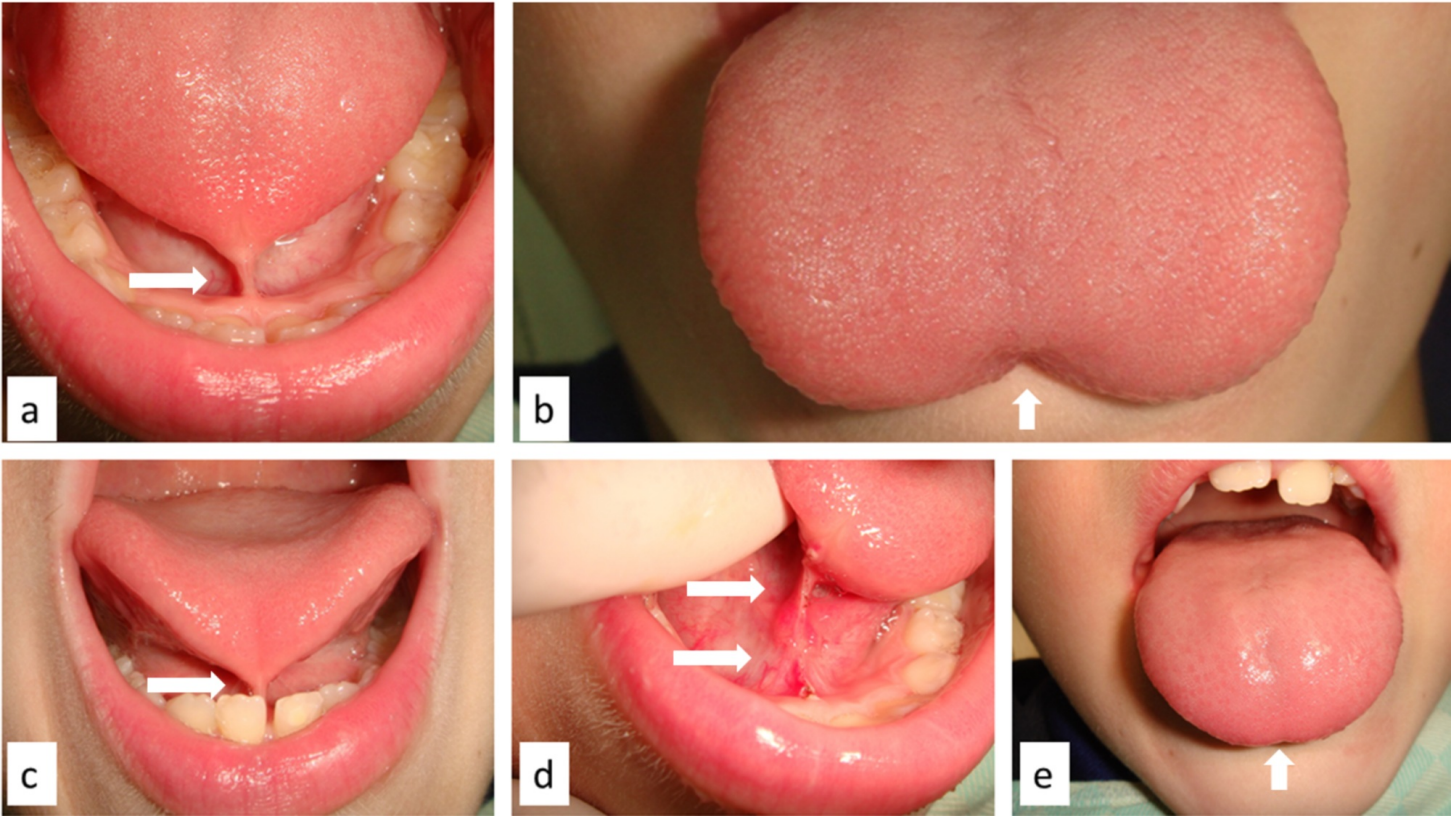
Tongue tip tied to the lingual frenulum and mandibular gingiva (a); classic W-shaped appearance of the tongue on protrusion (b); functional reduction of lingual movements (c); tongue frenulum appearance after diode laser surgery (d); immediate improvement of lingual movements (e).

A seven-year-old male patient was referred to us for difficulties in chewing and talking related to reduced tongue movement. Intraoral examination revealed severe ankyloglossia with the tongue tip tied to the lingual frenulum and gingiva of the lingual aspect of the mandible (Figure 1a), with a classic W-shaped appearance on tongue protrusion (Figure 1b). Tongue movement was minimal (Figure 1c), and the patient had difficulty in pronouncing some letters, especially “r” and “l.” Diode laser surgery of the frenulum was proposed to the child’s parents; conscious sedation was unnecessary as the patient seemed cooperative. With local infiltration of anesthesia, the frenulum was cut by diode laser (wavelength 800 ± 10 nm; continuous wave, output energy 1 Watt; Figure 1d), and tongue movements immediately improved (Figure 1e). Bleeding was absent during the procedure, stitches were unnecessary, postsurgical pain and edema were significantly reduced, and complete mucosal healing occurred within 10 days. Logopedic therapy was suggested to better correct speech and swallowing.

Case 2

**Figure 2:**
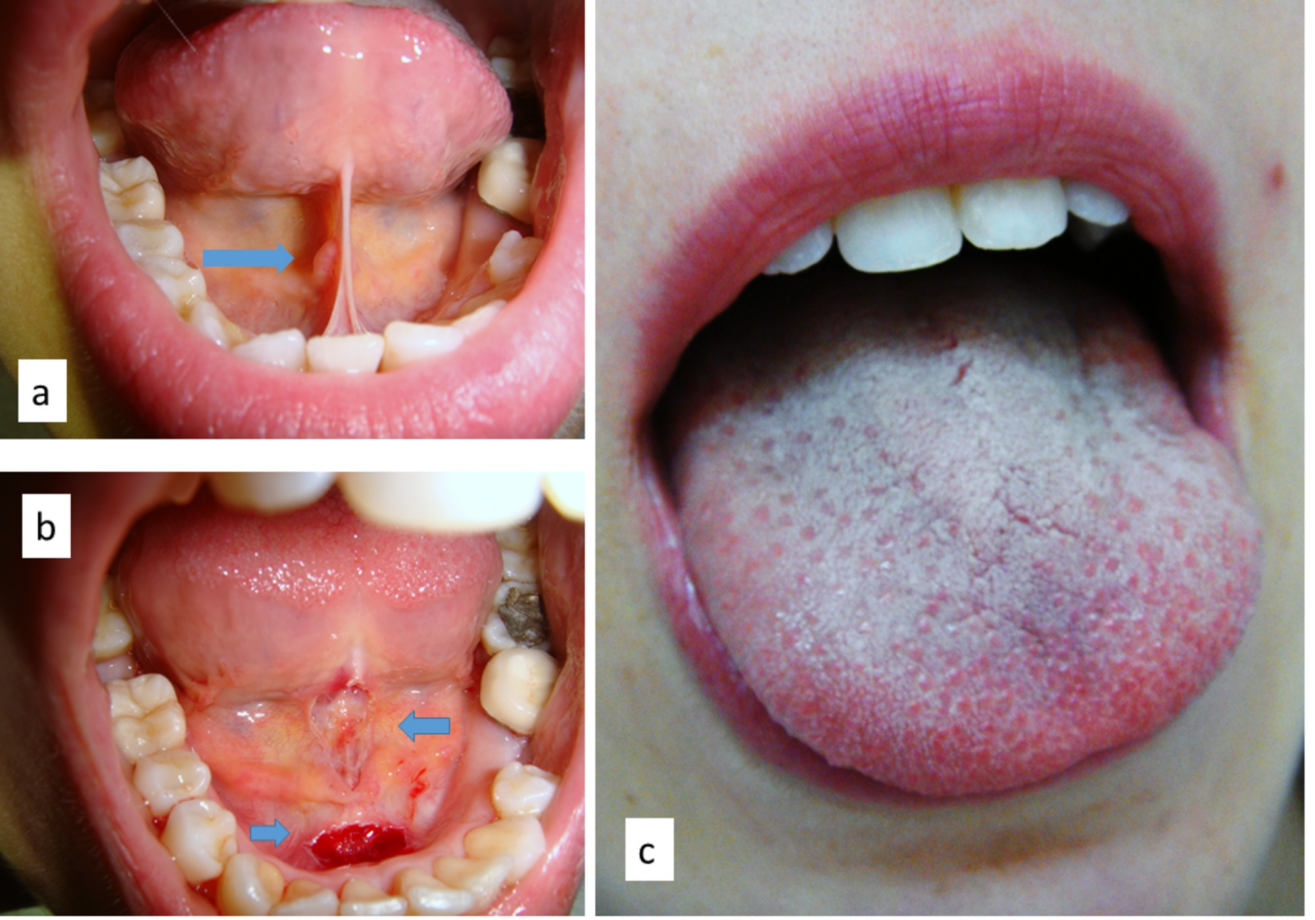
Thin and very short frenulum in a young girl (a), surgically cut by diode laser anterior and posterior (arrows) to the caruncula sublingualis (b); lingual movement improvement after treatment (c).

A 26-year-old female patient was referred for surgical treatment of the tongue frenulum for orthodontic purposes. Her medical history was uneventful, but she referred to dental anxiety. Intraorally, a thin but extremely short tongue frenulum was observable with a large insertion on the adherent gingiva, causing limitation of lingual movements (Figure 2a). A frenotomy with diode laser along with light conscious sedation was suggested, and the patient agreed. After conscious sedation and with minimal infiltration of local anesthesia, the frenulum was cut by diode laser (wavelength 800 ± 10 nm; continuous wave, output energy 1 Watt) both anterior and posterior to the caruncula sublingualis, without bleeding or the need for closure with stitches (Figure 2b). Postsurgical recovery was free of complications and restored tongue mobility. Surgical wounds completely healed within 14 days.

## Discussion

Several oral functions may be affected by tongue movement restriction related to ankyloglossia, such as sucking, swallowing, speech, chewing, and articular movements [4-6]. Therefore, early detection of a thick or very tight frenulum with or without limitation of lingual movements is essential especially at birth or in childhood [1-3,6]. In the past, conventional surgical approaches (with cold blade and stitches) surely represented a limitation for tongue-tie treatment both in adults and children as they were quite invasive and associated with intraoperative bleeding, postoperative edema, and functional limitation of tongue movements for at least one to two weeks after surgery [7,8]. Diode laser changed treatment approaches, due to laser capabilities of cut and contextual coagulation, the absence of unnecessary stitches, and faster healing of the oral mucosa [7-9]. In fact, among all lasers with proven surgical capability, the diode laser is the most used for surgical excision of proliferating (benign and malignant) lesions in the oral cavity, photocoagulation of vascular malformations, and nonsurgical periodontal treatment [10-13]. These clinical advantages become even more evident in the treatment of children with ankyloglossia, greatly increasing their acceptability [7]. This is also applicable in young adults suffering dental anxiety.

Additionally, operative time with diode laser is extremely reduced compared to conventional surgery [7,9]. In the case of very uncooperative patients, light conscious sedation may reduce dental anxiety in children or adults and facilitate the procedure [14,15]. Lastly, diode laser surgery, characterized by the total absence of intraoperative bleeding, is preferable in patients who may be affected by contagious infectious diseases as it reduces the risk of contagion and minimizes the risk of any related discrimination towards infected patients [16,17].

## Conclusions

As demonstrated by the described cases, diode laser surgery represents the more fitting treatment modality for ankyloglossia in patients of all ages as it is safe, noninvasive, and decisive, without major complications and high acceptance by parents and young patients. In addition, performing such surgical treatments by diode laser is relatively easier for oral surgeon than conventional scalpel surgery, and this is surely related to the intrinsic properties of the light laser itself. In fact, the possibility to cut and coagulate at the same time without causing thermal damages to the irradiated tissues, which usually results in a delayed healing, is the main characteristic of the diode laser use in oral surgery.
